# Relationship between Neutrophil-to-Lymphocyte Ratio and Systemic Lupus Erythematosus: A Meta-analysis

**DOI:** 10.6061/clinics/2020/e1450

**Published:** 2020-04-13

**Authors:** Liping Wang, Chunyan Wang, Xuqiang Jia, Minghui Yang, Jing Yu

**Affiliations:** I Vasculocardiology Department, Lanzhou University Second Hospital, Lanzhou University Second Clinical Medical College, Lanzhou University, Lanzhou 730030, China

**Keywords:** Systemic Lupus Erythematosus, NLR, Lupus Nephritis, Meta-Analysis

## Abstract

Systemic lupus erythematosus (SLE) is a chronic systematic autoimmune disease. Current methods of diagnosing SLE or evaluating its activity are complex and expensive. Numerous studies have suggested that neutrophil-to-lymphocyte ratio (NLR) is closely correlated with the presence of SLE and its activity, suggesting that it may serve as a diagnostic and monitoring indicator for SLE. Therefore, we performed a meta-analysis to systematically assess the association between NLR and SLE.

We performed a literature search until 12 April 2019 in the PubMed, Web of Science, and China National Knowledge Infrastructure databases. Cross-sectional studies comparing the NLR of SLE patients *versus* those of healthy controls, of active *versus* inactive SLE patients, and of SLE patients with *versus* without lupus nephritis were considered for inclusion. Mean intergroup NLR differences were estimated using standardized mean differences and their 95% confidence intervals. Study quality was assessed using the Agency for Healthcare Research and Quality instrument for cross-sectional studies.

Fourteen studies with 1,781 SLE patients and 1,330 healthy controls were included in this meta-analysis. The pooled results showed that the NLR was significantly higher in SLE patients than in healthy controls, in active SLE patients than in inactive SLE patients, and in SLE patients with lupus nephritis than in those without lupus nephritis.

NLR may be an indicator for monitoring disease activity and reflecting renal involvement in SLE patients. Nevertheless, more high-quality studies are warranted to further validate our findings.

## INTRODUCTION

Systemic lupus erythematosus (SLE) is a chronic systematic autoimmune disease with high heterogeneity 1,2. SLE is more prevalent in women of reproductive age than in men 3-5. Although the etiology and pathogenesis of SLE have not been fully elucidated, the breakdown of immune tolerance induced by environmental stimulation and genetic factors is widely reported to play a core role in SLE 1,2,6,7. The typical characteristics of SLE mainly include autoantibody production, aberrant complement system activation, immune complex deposition, and chronic inflammation, usually leading to multiple organ damage as well as diverse clinical manifestations 1,2,6,7.

The disease activity of SLE is related to its distinct pathogenesis and diverse clinical manifestations; thus, the treatment of SLE should be timely adjusted according to the changes in disease activity 2. The Systemic Lupus Erythematosus Disease Activity Index 2000 (SLEDAI-2K) score is most commonly applied to assess SLE disease activity 8. However, this score model incorporates both objective laboratory testing indicators and physician evaluation parameters 8; thus, it involves a certain degree of subjectivity. Additionally, the SLEDAI-2K score is complex and expensive, thereby inconvenient for clinical use. However, it holds great significance in identifying reliable, practical, and economical biomarkers to assist in diagnosing SLE and quantifying the disease activity of SLE patients. The immune system of SLE patients is abnormally activated by autoantigens, which results in immune complex deposition and complement system activation, followed by chronic inflammation 1,2,6,7,9. Furthermore, chronic inflammation stimulated by environmental and genetic factors is a common characteristic of autoimmune diseases including SLE 10,11. Several inflammatory markers, such as C-reactive protein, erythrocyte sedimentation rate, and interferon, have consistently been confirmed to be associated with SLE progression.

Peripheral blood neutrophil-to-lymphocyte ratio (NLR) is widely reported to be associated with inflammatory response and reflect the inflammatory status of many diseases 12-15. Moreover, numerous studies have also reported that NLR was closely related to SLE. Nevertheless, in these previous studies, several factors including small sample sizes, different methodologies, and a single-center setting may largely limit the reliability of their results. Therefore, here we performed a meta-analysis by combining the relevant data of these previous studies to comprehensively assess the association between SLE and NLR.

## METHODS

This meta-analysis adhered to the Preferred Reporting Items for Systematic Reviews and Meta-Analyses statement 16 and received approval from the Ethics Committee of Lanzhou University Second Hospital.

### Search strategy

We performed a comprehensive literature search of the PubMed, Web of Science, and China National Knowledge Infrastructure (CNKI) databases for relevant articles published up to 12 April 2019. The keywords used in the literature search included “systemic lupus erythematosus OR SLE” AND “lymphocyte” AND “neutrophil.” The search strategy used in PubMed was: (((lymphocyte [Title/Abstract]) AND neutrophil [Title/Abstract])) AND ((systemic lupus erythematosus [Title/Abstract]) OR SLE [Title/Abstract]). We searched for only studies published in English and Chinese since we understand only these two languages. The references in the identified studies were manually screened to identify other potential studies.

### Inclusion and exclusion criteria

The eligible studies included in this meta-analysis met all of the following criteria: (1) cross-sectional studies that compared the values of NLR between patients with SLE and healthy controls, patients with active SLE and those with inactive SLE, or SLE patients with lupus nephritis and those without lupus nephritis; (2) NLR data were presented as mean ± standard deviation; (3) if overlapped patients were enrolled in more than one studies, only the latest paper was considered in our meta-analysis.

The exclusion criteria were as follows: (1) reviews, editorials, case reports, letters, and conference abstracts; (2) duplicate publications; (3) mean ± standard deviation of NLR values not provided or could not be extracted by relevant information; and (4) unrelated topics.

### Data extraction

Two authors extracted the data independently, and any disagreement was resolved by discussion among all authors. The extracted data included name of first author, publication year, country, study type, case number, mean age, sex ratio, SLEDAI-2K score, SLE activity ratio, rate of lupus nephritis, SLE diagnostic criteria, therapy, and mean ± standard deviation of NLR values. The methodological quality of the included studies was assessed using the Agency for Healthcare Research and Quality instrument for cross-sectional studies 17. This instrument included 11 items assessed using “YES,” “No,” or “Unclear”: 1) Define the source of information (survey, record review); 2) List inclusion and exclusion criteria for exposed and unexposed subjects (cases and controls) or refer to previous publications; 3) Indicate time period used to identify patients; 4) Indicate whether the subjects were consecutively enrolled if the study was not population-based; 5) Indicate if the evaluators of the subjective study components were masked to the patients' other aspects; 6) Describe any assessments performed for quality assurance purposes (e.g., test/retest of primary outcome measurements); 7) Explain any patient exclusions from the analysis; 8) Describe how confounding variables were assessed and/or controlled for; 9) If applicable, explain how missing data were handled in the analysis; 10) Summarize patient response rates and completeness of the data collection; and 11) Clarify what follow-up, if any, was expected and the percentage of patients for whom incomplete data or follow-up were obtained. If the included studies only presented the median and range of NLR and sample size, we would estimate the mean ± standard deviation value by referring to the study by Hozo et al. 18.

### Statistical analysis

The mean NLR differences between patients with SLE and healthy controls, patients with active SLE and those with inactive SLE, and SLE patients with lupus nephritis and those without lupus nephritis were calculated using the standardized mean difference (SMD) and its 95% confidence intervals (95% CI) and visually described using a forest plot. When the 95% CI did not contain 0 and the SMD was simultaneously more than 0, the mean NLR relatively increased in patients with SLE, those with active SLE, or those with SLE and lupus nephritis. The chi-square test and I^2^ statistic were used to assess the heterogeneity among the included studies, while an I^2^ equal to or less than 50% signified no significant heterogeneity 19. The fixed-effect model would be applied for synthesized analysis if no significant heterogeneity existed (*p*>0.05) across the included studies; otherwise, a random-effects model was applied. Publication bias was visually assessed using a funnel plot produced from Begg's linear regression test, and Egger's linear regression test was further conducted to explore funnel plot asymmetry 20,21. The sensitivity analysis performed by sequentially omitting individual studies was used to explore whether the synthesized results were stable and reliable, while subgroup analyses were conducted to detect the sources of heterogeneity according to region and sample size. All statistical analyses were performed by Stata 12.0 software.

## RESULTS

### Study selection

**Figure 1:**
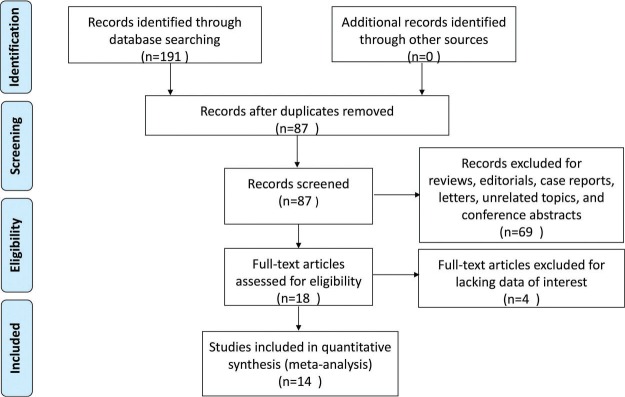
Flow diagram of the study selection process.

A flow chart of the study selection process is shown in Figure 1. A total of 191 potentially records were yielded through searches of PubMed, Web of Science, and CNKI. Among the total records, there were 104 duplicates, which were removed. Another 69 records were excluded owing to being reviews, editorials, case reports, letters, unrelated topics, or conference abstracts; as a result, 18 studies were left for full-text review. During the full-text review process, four studies were further excluded for lacking the data of interest. Finally, 14 studies with 1,781 SLE patients and 1,330 healthy controls were included in this meta-analysis 22-35.

### Study characteristics

All included studies were cross-sectional studies published between 2013 and 2019. The number of the enrolled patients and healthy controls in the included studies ranged from 21 to 344 and 30 to 170, respectively. Among the included studies, 11 were from China, one from Turkey, one from Indonesia, and one from Egypt. Overall, the methodological quality of the included studies was acceptable, although some items of the included studies were unclear. Among the included studies, 13 applied American College of Rheumatology classification criteria (ACR) to diagnose SLE, while the other applied ACR and Systemic Lupus International Collaborating Clinics criteria. More information regarding the main characteristics of the included studies is presented in Table 1. Among the included studies, 13 compared NLR between patients with SLE and healthy controls (Table 2), five compared NLR between patients with active SLE and those with inactive SLE (Table 3), and four compared NLR between SLE patients with and those without lupus nephritis (Table 4).

### Synthesized analysis

**Figure 2:**
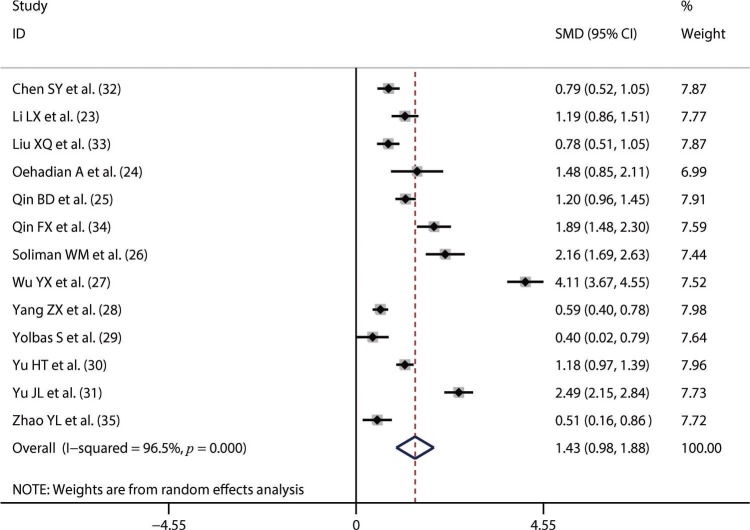
The neutrophil-to-lymphocyte ratio was significantly higher in systemic lupus erythematosus patients than in healthy controls. SMD, standardized mean difference.

**Figure 3:**
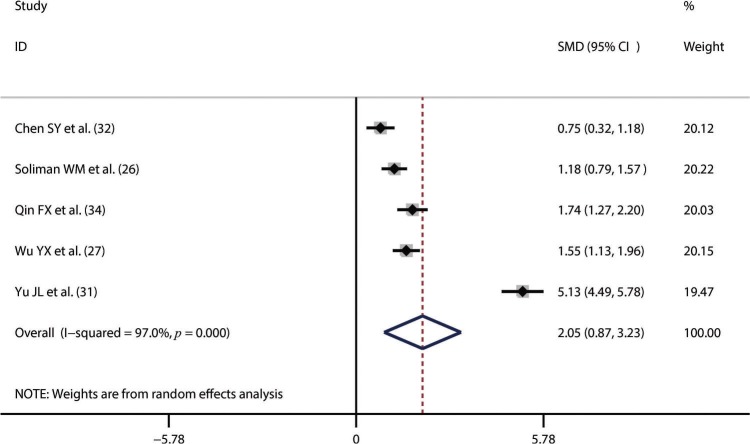
An increased neutrophil-to-lymphocyte ratio was closely associated with active systemic lupus erythematosus. SMD, standardized mean difference.

**Figure 4:**
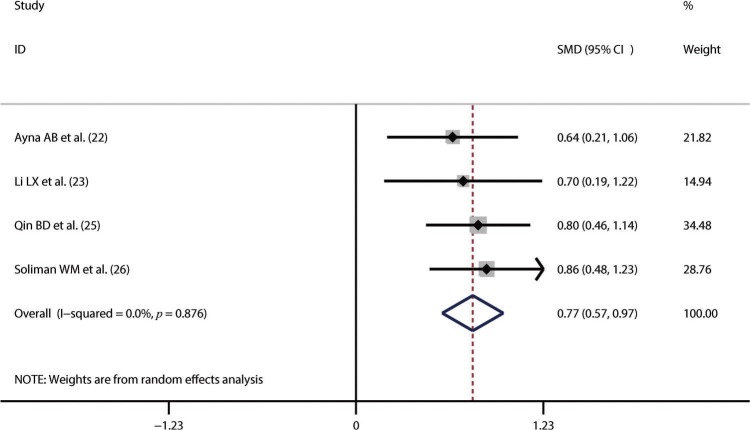
The neutrophil-to-lymphocyte ratio was significantly higher in systemic lupus erythematosus patients with lupus nephritis than in those without it. SMD, standardized mean difference.

Thirteen studies including 1,673 SLE patients and 1,330 healthy controls compared NLR between SLE patients and healthy controls 23-35. We performed the synthesized analysis of these studies using a random-effects model considering the significant heterogeneity across these studies (I^2^=96.5%, *p*<0.001). The synthesized result showed that NLR was significantly higher in SLE patients than in healthy controls (SMD=1.43; 95% CI, 0.98–1.88) (Figure 2). Five studies of 697 patients compared NLR between patients with active SLE and those with inactive SLE 26,27,31,32,34. The synthesized analysis of the five studies was also performed using a random-effects model due to the significant heterogeneity (I^2^=97.0%, *p*<0.001). The synthesized result showed that an increased NLR was closely associated with active SLE (SMD=2.05; 95% CI, 0.87–3.23) (Figure 3). Additionally, four studies of 441 patients explored the difference in NLR between SLE patients with *versus* without lupus nephritis 22,23,25,26. Considering the lack of significant heterogeneity (I^2^=0.0%, *p*=0.876), we conducted the synthesized analysis of the four studies using a random-effects model. The synthesized result showed that NLR was also significantly higher in SLE patients with *versus* those without lupus nephritis (SMD=0.77; 95% CI, 0.57–0.97) (Figure 4).

### Subgroup and meta-regression analysis

We performed the subgroup analysis and meta-regression according to region and sample size to explore the source of heterogeneity of the synthesized result of the NLR between SLE patients and healthy controls. The subgroup analysis results showed that significant heterogeneity still existed in each subgroup, which suggested that region and sample size may not be the main sources of heterogeneity (Table 5). Moreover, the results of meta-regression by region *(p*=0.65) and sample size *(p*=0.61) further confirmed that region and sample size were not mainly responsible for the heterogeneity (Table 5). Although we failed to identify the main sources of heterogeneity, we found that NLR was still significantly higher in SLE patients than in healthy controls in each subgroup (Table 5), indicating that our overall synthesized results were robust. Considering that the number of eligible studies was limited, we did not perform a subgroup analysis of the synthesized results of the relationship between NLR and disease activity as well as NLR and lupus nephritis.

### Sensitivity analyses and publication bias

**Figure 5:**
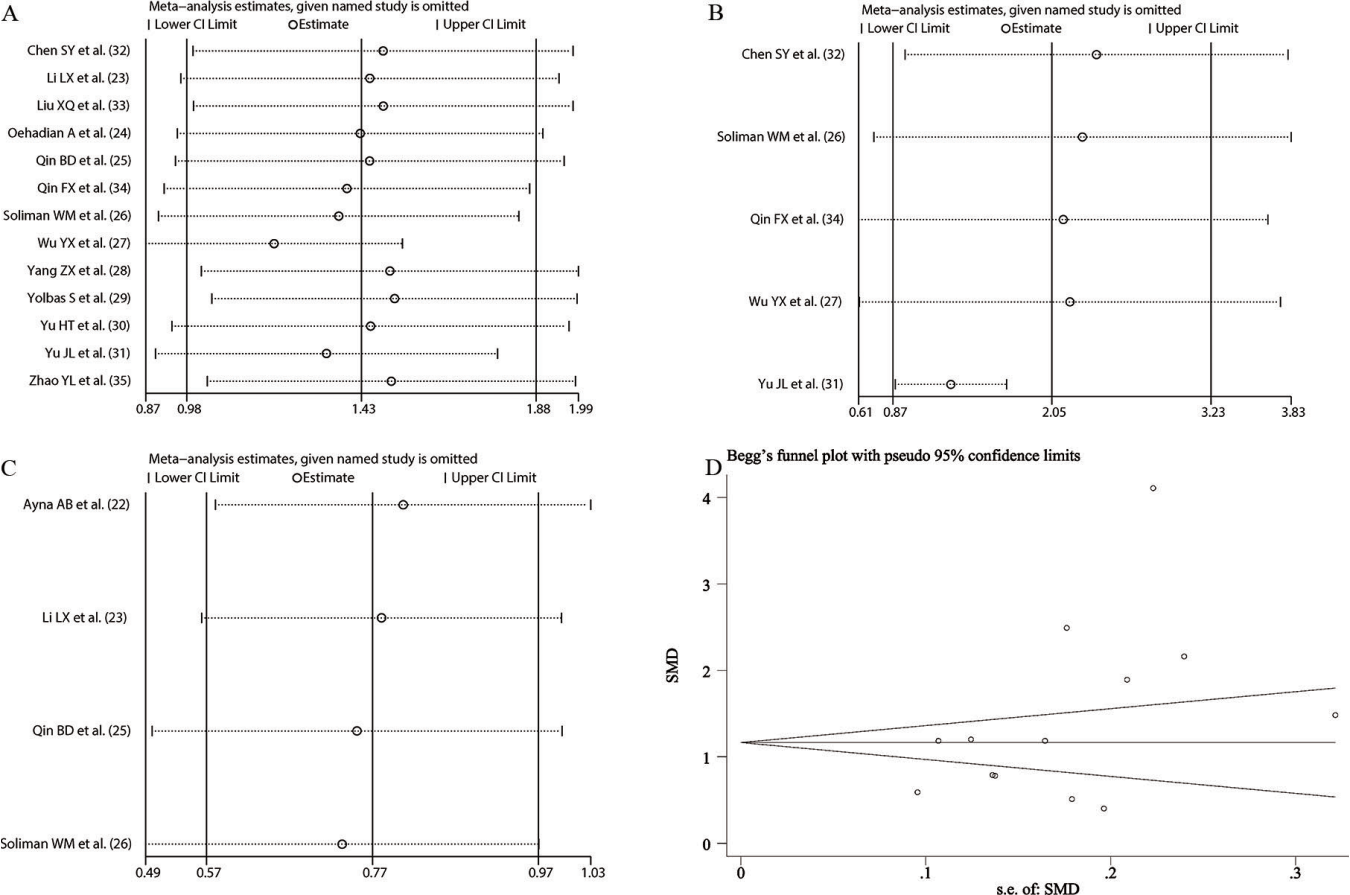
Sensitivity analysis of the relationship between neutrophil-to-lymphocyte ratio (NLR) and systemic lupus erythematosus (A), NLR and disease activity (B), and NLR and lupus nephritis (C). Begg's funnel plot used to assess potential publication bias in this meta-analysis (D). SMD, standardized mean difference.

To assess the stability of our synthesized results, a sensitivity analysis was performed by sequentially omitting individual studies and checking the consistency of the overall effect estimate. The results showed that the overall effect estimates of the relationship between NLR and SLE, NLR and disease activity, and NLR and lupus nephritis did not significantly change when any individual study was omitted (Figure 5A-5C). The publication bias evaluation was performed by the Egger's test and described graphically using the funnel plot produced from Begg's test. In our meta-analysis, the funnel plot was basically symmetrical and the *p* value of Egger's test was >0.05, suggesting that there was no significant publication bias in the synthesized result comparing NLR between SLE patients and healthy controls (Figure 5D). Therefore, our overall synthesized results were stable and reliable. Considering that the number of eligible studies was less than 10, we did not perform a publication bias assessment for the synthesized result about the relationship between NLR and disease activity as well as NLR and lupus nephritis.

## DISCUSSION

To our knowledge, no prior meta-analysis specifically assessed the association between SLE and NLR. Although numerous studies have reported that NLR was closely related to SLE, several factors including small sample sizes, different methodologies, and single-center settings may largely limit the reliability of their results regarding the association between SLE and NLR. Therefore, here we performed a meta-analysis by combining relevant data of these previous studies to comprehensively assess the association of SLE with NLR. In this meta-analysis, our overall synthesized results showed that NLR was significantly higher in SLE patients than in healthy controls, in patients with active *versus* inactive SLE, as well as in SLE patients with *versus* without lupus nephritis. Furthermore, our subgroup and sensitivity analyses demonstrated the robustness and reliability of these overall synthesized results.

Chronic inflammation stimulated by environmental and genetic factors is a common characteristic in most autoimmune diseases including SLE 10,11. The immune system of SLE patients is aberrantly activated by autoantigens, resulting in immune complex deposition, complement system activation, and chronic inflammation 1,2,6,7. Additionally, several inflammatory markers, such as C-reactive protein, erythrocyte sedimentation rate, and interferon, are positively associated with SLE progression. Therefore, there is a strong relationship between chronic inflammation and SLE. The inflammatory response is always accompanied by an increased release of many cytokines and chemokines, which are proven to intensify the immunoreaction and largely contribute to the pathogenesis of SLE 36-39. In particular, white blood cells (WBCs) are involved in the secretion of the cytokines and chemokines, which in turn activate these WBCs 40,41. Circulating WBCs and their classification counts will change, typically leading to lymphopenia and neutrophilia, in the presence of systemic inflammation 42,43. NLR is closely associated with most common inflammatory biomarkers, such as C-reactive protein and erythrocyte sedimentation rate 44-49. A growing body of evidence suggests that NLR may act as excellent indicator of inflammation status in various diseases, such as Crohn's disease 49, cancer 50,51, infection 52,53, and rheumatic diseases 54-56. Moreover, numerous studies also reported that NLR was closely related to inflammatory response and SLE disease activity 23-35. Consistent with these previous studies, in this meta-analysis, we further demonstrated a close correlation of NLR with disease activity and lupus nephritis in SLE patients. NLR can be easily measured, is simple, is rapidly reproducible, and is an economical biomarker. It is noteworthy that NLR has higher stability and reliability than individual blood cell parameters, since any individual blood cell parameter is easily affected by many factors such as dehydration, overhydration, and discrepancies in blood specimen handling 57,58. Therefore, NLR could be a dependable indicator for monitoring disease activity and reflecting renal involvement in SLE patients.

Several limitations of our meta-analysis should be seriously considered when interpreting our findings. First, there was significant heterogeneity. Although we performed the subgroup analysis and meta-regression according to sample size and region, we failed to identify the sources of heterogeneity. The differences in clinical complications, disease activity, disease duration, and therapies of SLE patients may cause significant clinical heterogeneity. Second, most of the included studies were from China; thus, it remains unclear whether our findings could be generalized to other populations, especially western countries. Third, our meta-analysis showed close associations of NLR with the presence of SLE, active SLE, or lupus nephritis. However, no relevant data in any of the included studies could be extracted for a synthesized analysis of the sensitivity and specificity of NLR for diagnosing SLE, disease activity, and lupus nephritis. Fourth, all included studies were retrospective and cross-sectional, which may have caused a degree of bias. Besides, due to design principles, a cross-sectional study cannot be used to analyze whether there is a causal correlation between a risk factor and a disease or its progression. Thus, the current study cannot identify whether NLR is a potential risk factor for SLE development. Certainly, cross-sectional studies could reflect a simple association of NLR with SLE at some point in time, suggesting that results from cross-sectional studies may help to explore the value of NLR in assisting the diagnosis and surveillance of SLE. Evidence from cross-sectional studies is weak; thus, more high-quality studies (prospective cohort or randomized) are required to further validate the association between NLR and SLE. Fifth, only studies published in English and Chinese were considered in this meta-analysis, with studies published in other languages excluded, which may also result in a degree of bias. Sixth, although this meta-analysis combined data of the published studies on this topic, the total sample size was still insufficient, which may affect the reliability of the combined results. Finally, any diagnostic biomarker should be evaluated for sensitivity and specificity. However, we did not perform a meta-analysis of the accuracy tests to assess the sensitivity and specificity of NLR for diagnosing SLE, active SLE, or lupus nephritis since we could not extract available data from the eligible studies. That is, the current study only suggested simple correlations between NLR and SLE, active SLE, or lupus nephritis and could not determine the diagnostic value of NLR. Hence, further studies are needed to validate the clinical value of NLR for diagnosing SLE, active SLE, or lupus nephritis.

In conclusion, this meta-analysis demonstrated that NLR was closely associated with SLE, suggesting that it may be a promising indicator for monitoring disease activity and reflecting renal involvement in SLE patients. However, more high-quality studies with large sample sizes are needed to further confirm our findings.

## AUTHOR CONTRIBUTIONS

Wang L and Yu j conceived and designed this meta-analysis. Wang L wrote the manuscript. Wang C and Jia X performed the literature search and extracted the data. Yang M conducted the statistical analysis.

## Figures and Tables

**Table 1:** Main characteristics of the included studies.

					Patients with SLE						Healthy controls			
Study	Region	Study type	No.	Age (Mean±SD)	Sex (F/M)	SLEDAI (Mean±SD)	With activity (%)	With LN (%)	SLE diagnostic criteria	Therapy	No.	Age (Mean±SD)	Sex (F/M)	NOS
Ayna AB et al. (22)	Turkey	R	108	35.3±10.2	100/8	NR	28	72	ACR	NR	NR	NR	NR	7
Chen SY et al. (32)	China	R	113	39.01±12.28	105/8	NR	26.5	NR	ACR	NR	120	37.58±13.64	112/8	7
Li LX et al. (23)	China	R	59	29.47±12.63	55/4	5.51±3.76	NR	0	NR	No	149	28.44±4.42	132/17	6
Liu XQ et al. (33)	China	R	127	37.86±15.70	113/14	14.87±7.85	NR	NR	NR	NR	103	41.01±12.60	87/16	7
Oehadian A et al. (24)	Indonesia	R	21	NR	21/0	NR	57.2	61.9	ACR	No	30	NR	20/10	6
Qin BD et al. (25)	China	R	154	41.44±14.56	137/17	8.17±5.66	NR	64.3	ACR	No	151	43.56±13.97	131/20	7
Qin FX et al. (34)	China	R	100	NR	54/46	NR	58	NR	ACR	NR	47	NR	47/22	6
Soliman WM et al. (26)	Egypt	R	120	29.93±8.72	102/18	NR	50	50	ACR	No	30	27.40±4.97	21/9	6
Wu YX et al.(27)	China	R	116	NR	97/19	11.39±6.48	NR	64.66	ACR	No	136	NR	111/25	7
Yang ZX et al. (28)	China	R	344	38±15	303/41	NR	NR	NR	ACR, SLICC	No	170	45±10	151/19	8
Yolbas S et al. (29)	Turkey	R	51	33±9.6	47/4	NR	NR	NR	ACR	NR	55	45.1±13	44/11	6
Yu HT et al. (30)	China	R	212	40.19±15.24	189/23	10.67±6.63	15.5	NR	ACR	No	201	41.45±12.08	181/20	8
Yu JL et al. (31)	China	R	194	40.61±12.50	179/15	NR	NR	NR	ACR	NR	71	43.24±13.09	61/10	7
Zhao YL et al. (35)	China	R	62	41±9	10/52	NR	NR	NR	ACR	NR	67	39±10	55/12	6

ACR, American College of Rheumatology; LN, lymph node; NR, not reported; R, ; SD, standard deviation; SLE, systemic lupus erythematosus; SLEDAI, Systemic Lupus Erythematosus Disease Activity Index; SLICC, Systemic Lupus International Collaborating Clinics

**Table 2:** Neutrophil-to-lymphocyte ratio of SLE patients *versus* healthy controls.

	Patients with SLE			Healthy controls		
Study	Case number	Mean	SD	Case number	Mean	SD
Chen SY et al. (32)	113	4.01	3.65	120	1.95	0.85
Li LX et al. (23)	59	4.26	3.38	149	2.00	0.76
Liu XQ et al. (33)	127	3.08	2.46	103	1.63	0.50
Oehadian A et al. (24)	21	4.24	2.48	30	1.65	0.96
Qin BD et al. (25)	154	3.61	2.04	151	1.82	0.49
Qin FX et al. (34)	100	4.94	2.11	47	1.47	1.01
Soliman WM et al. (26)	120	3.16	1.00	30	1.21	0.21
Wu YX et al. (27)	116	2.77	0.38	136	1.64	0.13
Yang ZX et al. (28)	344	3.05	2.70	170	1.72	0.75
Yolbas S et al. (29)	51	2.90	4.15	55	1.7	1.13
Yu HT et al. (30)	212	3.66	1.91	201	1.99	0.49
Yu JL et al. (31)	194	3.61	0.37	71	2.80	0.14
Zhao YL et al. (35)	62	2.59	2.55	67	1.63	0.87

SD, standard deviation; SLE, systemic lupus erythematosus

**Table 3:** Neutrophil-to-lymphocyte ratio in SLE patients with active *versus* inactive SLE.

	Patients with active SLE			Patients with inactive SLE		
Study	Case no.	Mean	SD	Case no.	Mean	SD
Chen SY et al., 2017	30	5.52	5.15	83	3.08	2.23
Soliman WM et al., 2018	60	3.88	1.33	60	2.21	1.50
Qin FX et al., 2018	58	6.32	1.28	42	4.06	1.33
Wu YX et al., 2016	64	3.25	0.70	52	2.34	0.41
Yu JL et al., 2018	30	5.71	1.26	164	3.11	0.14

SD, standard deviation; SLE, systemic lupus erythematosus.

**Table 4:** Neutrophil-to-lymphocyte ratio of SLE patients with *versus* without nephritis.

	SLE patients with nephritis			SLE patients without nephritis		
Study	Case no.	Mean	SD	Case no.	Mean	SD
Ayna AB et al., 2017	78	5.9	5.9	30	2.6	2.5
Li LX et al., 2015	20	7.21	6.01	59	4.26	3.38
Qin BD et al., 2016	99	4.10	1.65	55	2.74	1.77
Soliman et al., 2018	60	4.27	1.74	60	2.86	1.54

SD, standard deviation; SLE, systemic lupus erythematosus.

**Table 5:** Subgroup analysis and meta-regression of the relationship between NLR and SLE.

			Heterogeneity		Meta-regression		
Stratified factors	No. of studies	Pooled SMD (95% CI)	I^2^ (%)	*p* value	Tau^2^	Adj R^2^ (%)	*p* value
Publication year					1.07	-6.24	0.58
>2017	4	1.64 (0.96-2.32)	97	<0.01			
≤2017	2	1.30 (0.70-1.91)	95.1	<0.01			
Sample size					1.08	-6.78	0.61
n≤214	6	1.26 (0.69-1.83)	91.5	<0.01			
n>214	7	1.58 (0.90-2.25)	97.9	<0.01			
Region					1.08	-7.34	0.65
China	10	1.46 (0.94-1.98)	97.10%	<0.01			
Indonesia	1	1.48 (0.85-2.11)	-	-			
Egypt	1	2.16 (1.69-2.63)	-	-			
Turkey	1	0.40 (0.02-0.79)	-	-			

CI, confidence interval; NLR, neutrophil-to-lymphocyte ratio; SLE, systemic lupus erythematosus; SMD, standardized mean difference.
